# From birth to death: The hardworking life of Paneth cell in the small intestine

**DOI:** 10.3389/fimmu.2023.1122258

**Published:** 2023-03-10

**Authors:** Chenbin Cui, Fangke Wang, Yao Zheng, Hongkui Wei, Jian Peng

**Affiliations:** ^1^ Department of Animal Nutrition and Feed Science, College of Animal Science and Technology, Huazhong Agricultural University, Wuhan, China; ^2^ The Cooperative Innovation Center for Sustainable Pig Production, Wuhan, China

**Keywords:** Paneth cell, cell differentiation, intestinal stem cell, antibacterial peptide, cell death

## Abstract

Paneth cells are a group of unique intestinal epithelial cells, and they play an important role in host-microbiota interactions. At the origin of Paneth cell life, several pathways such as Wnt, Notch, and BMP signaling, affect the differentiation of Paneth cells. After lineage commitment, Paneth cells migrate downward and reside in the base of crypts, and they possess abundant granules in their apical cytoplasm. These granules contain some important substances such as antimicrobial peptides and growth factors. Antimicrobial peptides can regulate the composition of microbiota and defend against mucosal penetration by commensal and pathogenic bacteria to protect the intestinal epithelia. The growth factors derived from Paneth cells contribute to the maintenance of the normal functions of intestinal stem cells. The presence of Paneth cells ensures the sterile environment and clearance of apoptotic cells from crypts to maintain the intestinal homeostasis. At the end of their lives, Paneth cells experience different types of programmed cell death such as apoptosis and necroptosis. During intestinal injury, Paneth cells can acquire stem cell features to restore the intestinal epithelial integrity. In view of the crucial roles of Paneth cells in the intestinal homeostasis, research on Paneth cells has rapidly developed in recent years, and the existing reviews on Paneth cells have mainly focused on their functions of antimicrobial peptide secretion and intestinal stem cell support. This review aims to summarize the approaches to studying Paneth cells and introduce the whole life experience of Paneth cells from birth to death.

## Introduction

The intestinal epithelium, which consists of a single layer of intestinal cells, is an important barrier separating intestinal contents from tissues (1). The intestinal epithelium is composed of absorptive cells such as enterocytes (the most abundant cell type) and secretory cells such as Paneth cells (PCs), goblet cells, and enteroendocrine cells (2). The small intestine is structurally divided into two parts, convex villus and concave crypts (3). Unlike other secretory cells, PCs, as a unique type of epithelial cells, are located at the bottom of crypts where they are intercalated between intestinal stem cells (ISCs).

PCs were first identified by Gustav Schwalbe based on the obvious cytoplasmic granules in 1872 (4), and further named by Josef Paneth in 1887 (5). PCs are normally observed in the small intestine, especially ileum, with 5-15 cells per crypt (6, 7). However, in pathological condition, PCs are detected at other tissues such as stomach and colon, and this phenomenon is called PC metaplasia (8). Although metaplastic PCs exhibit a protective effect against infections, they also promote tumorigenesis by secreting growth-promoting factors (8).

In contrast to the 4-5 day lifespan of enterocytes, PCs survive approximately one month in crypts (9). PCs are widely found in the intestine of various species such as humans, mice, pigs, and horses (10–12). However, most studies on PCs are conducted based on the samples from humans and mice, and the results have shown that the development of PCs in humans is different from that in mice. The first emergence of PCs in humans is before birth (13.5 weeks gestational age), while that in mice is after birth (7-10 days old) (13, 14). However, the development and functions of PCs in other species remain largely unclear (12).

Recently, PCs have attracted many researchers due to their beneficial effects on the intestine (1), and recent research highlights the importance of PCs in the regulation of intestinal microbiota and ISCs. To promote a comprehensive understanding of PCs, this review discusses the main materials and methodology for PC study and the events during entire lives of PCs.

## Morphological characteristics of PCs

The presence of abundant granules in the cytoplasm is the first clue to PC discovery, and it remains a histological feature used for PC identification now (4). These granules in the apical cytoplasm of PCs mainly contain multiple antibacterial peptides (AMPs) such as lysozyme, α-defensin, secretory phospholipase A2 (sPLA2) and some proteins such as lipopolysaccharide-binding protein (LBP) and norepinephrine (9, 15, 16). The discovery of lysozyme in the granules is the first evidence of the protective role of PCs in the intestine (17). PCs possess extensive endoplasmic reticulum (ER) and trans-Golgi network (TGN) to synthesize and process proteins, which enables them to rapidly supplement the released granules (9). The above morphological characteristics suggest that PCs are a unique type of intestinal epithelial cell with particular structure. Notably, staining identification shows that PCs of some species such as pigs contain no granules (18).

## Materials and methods for PC investigation

Multiple materials and methods are required to investigate the alterations of PCs. Selecting appropriate experimental materials is important for PC research. The majority of PC-related studies utilize C57BL/6 mouse strain since this mouse strain possesses more PCs with an abundant AMP profile, compared to 129/SvEv mouse strain (19).

In PC studies, PC interference tends to be conducted by chemical and transgenic methods in mice (20, 21). Dithizone, a zinc chelator, has been reported to selectively eliminate PCs from the small intestine of mice and rats (22). However, the PC-ablation effect of dithizone lasts only for a short time, and then renascent PCs emerge in the crypts at 12 h after ablation and completely restore to normal state at 72 h (22). Intestinal transforming growth factor (TGF)-α and TGF-β_1_ are responsible for the rapid regeneration of PCs (23). Another method for PC ablation includes two steps: first, a human diphtheria toxin (DTX) receptor is inserted into mouse’s cryptdin-2 (*Defa6*) promoter to produce transgenic mouse strain PC-DTR (20), and then administration of PC-DTR mice with DTX leads to a decline of PC number (20, 24). Nevertheless, these two methods exhibit different effects in many aspects. In terms of PC-ablation efficiency, lysozyme staining results show that dithizone can reduce PCs by 33%, while DTX administration can reduce PCs by 60% (25). In addition, dithizone also contributes to autophagy-like changes in PCs (25). Tumor necrosis factor (TNF)-α released from dithizone-induced degenerated PCs can activate NF-κB signaling, thus promoting intestinal cell proliferation in rats (26). Furthermore, PC disruption induced by dithizone, rather than by DTX, impairs the small intestinal perfusion by down-regulating nitric oxide signaling (27). DTX administration leads to PC necrosis, thus possibly causing an increase in serum interleukin (IL)-6, IL-10, and TNF, whereas dithizone fails to trigger intestinal inflammation (25). These studies suggest that dithizone and DTX may act on PCs *via* different mechanisms. Therefore, it is necessary to make the appropriate choice between dithizone and DTX to delete PCs according to the objective of research. Additionally, gene editing makes it convenient to study the influence of certain gene on PCs in C57BL/6 mice. Mice with genes atonal homolog 1 (*Atoh1*), sex determining region Y-box 9 (*Sox9*), or growth factor independent 1 transcription repressor (*Gfi1*) knocked out are usually used as a PC ablation model (28–30). In addition to the methods of complete PC disruption mentioned above, transgenic *Defa6*-cre mice have been used to generate PC-specific gene knockout mice, which allows the exploration of the functions of certain gene in PCs (31). Notably, transgenic *Defa6*-cre mouse strain is established by Professor Blumberg (31), and he generously shares this mouse strain with other researchers (32, 33).

With the exception of mice, intestinal organoids, which are similar to the physiological structure and function of the intestinal epithelium *in vivo*, are also utilized to study PCs (34, 35). The intestinal organoids with a three-dimensional structure contain various intestinal epithelial cells formed from the continuous differentiation of ISCs *ex vivo*. Hans Clevers and Toshiro Sato first established intestinal organoids based on isolated ISCs in 2009, which lays a foundation for subsequent research on intestinal organoids (34). PCs intermingled with ISCs also exist at the bottom of crypts in organoids. Compared with mouse models, the intestinal organoids make PC studies more convenient, exhibiting multiple advantages such as stability and controllability.

Using various methods such as qPCR, western blot, and immunostaining, the alterations of PCs are examined in the mouse models and intestinal organoids (36, 37). Among them, histochemical staining mainly including periodic acid Schiff’s staining, eosin and phloxine-tartrazine stainings can visualize the abundant granules in the cytoplasm of PCs (38) In addition, transmission electron microscopy (TEM) enables researchers to observe the intracellular components of PCs such as granules and ER (33). Considering that PCs can express several unique genes (*Defa1*, *Defa5*, and *Lyz1*), the detection of these PC markers using qPCR, RNA-seq, and fluorescence *in situ* hybridization (FISH) usually reflects the changes of PCs at the gene expression level (11, 39). The western blot, immunohistochemistry, and immunofluorescence assays of α-defensin and lysozyme reveal the changes of PCs at the protein level. Notably, it is necessary to examine these PC markers at both mRNA and protein levels since the results at these two levels might be inconsistent due to the translational block caused by unfolded protein response (UPR) activation (40).

Flow cytometry (FACS) with CD24 antibody is used to analyze the PC number (11). FACS in combination with cell sorting allows the isolation of live PCs from the intestine (32). The proportion of PCs in CD24^+^ cells is extremely low (approximately 1.87%) (32). The isolated PCs can be cultured *in vitro*, which enables researchers to observe the alterations in PCs directly under different treatments (32). Another method for investigating molecular changes of PCs is laser capture microdissection (LCM). LCM can precisely isolate PCs from embedded tissue sections without impairing tissue structure for subsequent analysis (32, 41). Compared with FACS, LCM is more precise since it reflects the real changes in PCs under physiological condition.

## Origin of PC life: Differentiation and migration

**Figure 1:**
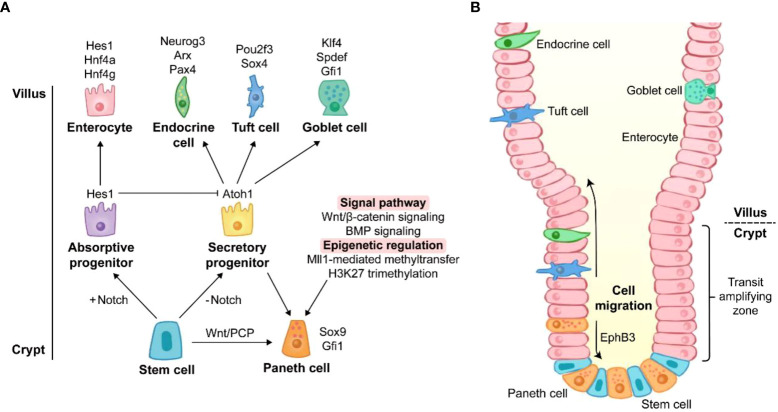
The origin of Paneth cell life. **(A)** Differentiation of Paneth cells. The differentiation towards secretory cells occurs when Notch signaling is off. Sox9 and Gfi1 are two key transcription factors of Paneth cell lineage. Wnt/PCP signaling directly drives the conversion of intestinal stem cells to Paneth cells. Wnt/β-catenin signaling and BMP signaling promote the lineage commitment of Paneth cells. Recent studies have reported that the differentiation of Paneth cells is also affected by epigenetic regulation such as Mll1-mediated methyltransfer and H3K27 trimethylation. The genes around the cell images are key transcription factors for the cell lineage commitment. **(B)** Migration of Paneth cells. Unlike other secretory cells, Paneth cells are located at the base of crypts. After differentiation, Paneth cells migrate downward to crypt bottom, and they are intercalated between stem cells. The migration of Paneth cells is mediated by EphB3.

Coexistence of multiple cell types in the intestinal epithelium suggests that cell differentiation is a strict and complex process (**Figure 1A**). Cell differentiation in the intestine is determined by several signaling pathways such as Notch, Wnt, and bone morphogenetic protein (BMP) signaling (2). PC differentiation is conducted under the conditions of Notch signaling off and Wnt signaling on.

Notch signaling is the key for lineage commitment towards absorptive cells or secretory cells. The 5-day Notch signaling inhibition by dibenzazepine (DBZ), an inhibitor of γ-secretase, leads to PC hyperplasia in mice (42). However, a recent study showed that acute Notch signaling inhibition (1 day after DBZ treatment) induced rapid apoptosis of PCs with a robust rebound of PCs at day 7 post DBZ treatment, suggesting the beneficial role of Notch signaling in PC maintenance rather than in lineage commitment (43). The dominance of Notch signaling in lineage commitment is achieved by its target gene, hairy and enhancer of split 1 (*Hes1*). *Hes1* directly suppresses the transcription of *Atoh1* which is responsible for the differentiation of secretory cells (42). Like DBZ-treatment, the deletion of *Hes1* promotes PC differentiation in the small intestine (44). Moreover, deletion of Notch ligands *Dll1* and *Dll4* and their receptors *Notch1* and *Notch2* also contributes to an increase in PCs (42, 45, 46). It should be noted that *Dll1* knockout is accompanied by the elevated expression of *Dll4* (45), and *Notch1* knockout induces the expression increase of *Dll1* and *Dll4* (46), which may be attributed to the increased PCs.

Wnt/β-catenin signaling can activate transcription factor 4 (Tcf4)-mediated transcription *via* nuclear localization of β-catenin, and this signaling plays a crucial role in PC differentiation in the intestine. Transgenic mice overexpressing *Dkk1* (a Wnt signaling inhibitor) exhibit the absence of all secretory cell lineages including PCs (47). In contrast, Apc^lox/lox^VilCreER^T2^ and Catnb^lox(ex3)^Vil-CreER^T2^ mice (mouse models of Wnt/β-catenin activation) display higher level of PC differentiation, compared with wild type (WT) mice (48). *Atoh1*, a downstream gene of Wnt/β-catenin signaling, is essential for the maintenance of secretory progenitors. High expression of *Atoh1* and nuclear localization of β-catenin/Tcf4 have been reported in PCs (47, 49). *Atoh1* deletion results in a decrease in all secretory cell number (50) and the conversion from specified secretory cells into functional enterocytes (51). Furthermore, other factors are also involved in driving the conversion from secretory progenitors to the PC lineage rather than other secretory cells. Sox9 and Gfi1 are the main transcription factors responsible for the differentiation of PCs. *Sox9* is also a target gene of Wnt/β-catenin signaling, and Gfi1 functions in the downstream of Atoh1 (29, 30). Lack of *Sox9* or *Gfi1* leads to PC lineage defects in mice (29, 30). Surprisingly, Atoh1 strengthens its own expression and directly regulates Dll1 and Dll4 which mediate lateral inhibition, a process maintaining a proper proportion of absorptive cells and secretory cells (51). Similar to canonical Wnt/β-catenin signaling, non-canonical Wnt/planar cell polarity (PCP) signaling is also involved in PC lineage differentiation, in spite of the mutual antagonization of their functions in some cases (52). Wnt/PCP signaling drives the differentiation from ISCs directly to PC and enteroendocrine cell lineages without the intermediate step of secretory progenitors (53).

In addition to Notch and Wnt signalings, several signaling pathways also act on PC lineage differentiation. BMP signaling modulates secretory cell maturation without interfering with Wnt/β-catenin signaling. The inhibition of BMP signaling by silencing BMP receptor type IA impairs terminal differentiation of PCs (54). Mammalian mitogen-activated protein kinase (MAPK) signaling disruption leads to a PC maturation defect (55). The nonreceptor tyrosine phosphatase Shp2-mediated MAPK signaling controls the fate between goblet cells and PCs, and Shp2/MAPK signaling weakening causes an increase in PC number meanwhile repressing goblet cell development (56).

In addition to transcription factors and signaling pathways, epigenetic regulation emerges as a novel modulator of PC lineage. The regulation of Wnt and MAPK signalings by histone methyltransferase Mll1 controls the lineage allocation between PCs and goblet cells (57). The inhibition of histone H3 lysine 27 (H3K27) trimethylation impairs PC maturation in the intestinal organoids (58). Thus, these studies indicate that PC lineage differentiation and development are intricate processes under the controls of multiple signaling pathways at both transcriptional and post-transcriptional levels.

After differentiation, most other cells migrate upward to villus, while PCs move to crypt bottom (**Figure 1B**) (59). Ephrin type-B receptor 3 (*EphB3*) is highly expressed in PCs and responsible for PC migration (60), and *EphB3-*knockout mice exhibit the disordered location of PCs along the villus-crypt axis (60). Since *EphB3* is also a downstream target of Wnt/β-catenin signaling, Wnt inhibition *via Frizzled-5* (a Wnt receptor on membrane) silencing and *Dkk1* ectopic expression results in random scatter of PCs, and the absence of EphB3 is found in the disordered PCs (47, 49). Therefore, Wnt signaling is of great importance for both differentiation and migration of PCs.

## PC functions in intestine

Located at the bottom of small intestinal crypts, PCs possess multiple functions such as shaping intestinal microbiota, promoting regeneration, and controlling inflammation. PC disruption by chemical and transgenic methods has a negative influence on the intestine as discussed above. AMPs secreted by PCs shape the structure of intestinal microbiota and confer crypts with a sterile environment. The ISC-supporting role of PCs facilitates intestinal regeneration *in vivo* and organoid formation *ex vivo*. Clearance of apoptotic cells from crypts by PCs prevents the occurrence of inflammation. Here we discuss the various participations of PCs in the intestinal homeostasis.

## AMP secretion

There are trillions of microorganisms in the intestinal lumen, most of which are harmless and commensal. These commensal bacteria protect the intestine against pathogenic bacteria and produce nutrients from the fermentation of intestinal contents (61). However, the pathogenic bacteria with strong virulence can colonize the intestinal epithelium to invade host and exacerbate infections. PCs, as a source of abundant AMPs, play an important role in both controlling composition of commensal bacteria and defending against pathogenic bacteria. AMPs packed in apical cytoplasmic granules are an ancient type of host defense effectors in the intestinal innate immunity. The high AMP secretory characteristics of PC makes it an essential part of the intestinal innate immune system. In addition, the involvements of PCs in the intestinal innate immunity are also demonstrated by their functional proteins such as LBP, MD-2, and integrin α6β4 (15, 62, 63). Therefore, PCs could be called innate immune cells. Recent evidence has revealed that there are two subtypes of PCs which are distinguished by the generation of fucosyltransferase 2 (Fut2) in mice (64). The major source of AMPs is the Fut2^+^ PCs mainly in ileum, rather than the Fut2^-^ PCs in duodenum, and Fut2^+^ PCs exhibit the higher granularity and structural complexity (64). PC-derived AMPs mainly include α-defensins (cryptdins in mice), lysozyme, secreted phospholipase A2 (sPLA2), and regenerating islet-derived 3α (REG3α, REG3γ in mice) (38). These AMPs exhibit similar bactericidal ability through bacterial binding and membrane perforation (65).

α-defensins account for 70% in all the AMPs secreted by PCs (66). Mammalian α-defensins exhibit a length of 30-40 amino acids with 6 cysteine residues forming disulfide bridges (38). Human PCs contain 2 α-defensins, HD5 and HD6, while mice PCs possess 6 cryptdin isoforms, cryptdin-1 to -6 (67). PC-specific cryptdin-1 and cryptdin-6 are transcriptionally regulated by β-catenin/Tcf4 signaling, and *Frizzled-5* conditional deletion in the intestinal epithelia abolishes the gene expression (49). The mouse model with PC ablation mediated by dithizone treatment or gene knockout exhibits the disorders in intestinal microbiota and the higher susceptibility to bacterial infections, compared to control mice (25, 33, 39). In addition, α-defensin misfolding in abnormal PCs triggers intestinal inflammation and instability of intestinal microbiota (68). Defa1 administration or adenovirus-induced HD5 expression rescues mice with PC defects from severe intestinal injury (39, 69). These studies highlight the important role of α-defensins secreted by PCs in the intestinal homeostasis maintenance.

Additionally, α-defensins do not display antibacterial ability until they are cleaved into their active forms after secretion, and this cleavage process is performed by trypsin in human and matrix metalloproteinase-7 (Mmp7) in mice (70, 71). *Mmp7* knockout mice display the alterations in the intestinal microbial community and develop more severe inflammation after infections (70, 72), suggesting the necessity of α-defensin maturation in the intestine. Moreover, the presence of high-level zinc (a cofactor of Mmp7) in PC granules is necessary for Mmp7 activity and AMP stabilization (70, 73, 74). Since zinc transporter 2 facilitates zinc import into PC granules, the ablation of this zinc transporter impairs PC functions and AMP secretion (75). Zinc supplementation alleviates PC necroptosis and intestinal microbiota disorder induced by TNF treatment in mice (76).

Another AMP is lysozyme, a widespread used PC marker in mice, and lysozyme is responsible for peptidoglycan hydrolyzation in the bacterial cell walls (38). There are two types of lysozyme in mice, *Lyz1* (expressed by PCs)-encoded lysozyme type P and *Lyz2* (expressed by macrophages)-encoded lysozyme type M (38). In human, lysozyme is not unique to PCs, and it is also present in BEST4 cells and follicle associated epithelium (FAE), as demonstrated by single-cell transcriptomic analyses involving duodenum, jejunum, ileum, and colon from three humans (77). Unexpectedly, *Lyz1* deficiency has been reported to result in a type 2 immune response to protect mice from DSS (dextran sodium sulfate)-induced colitis *via* IL-13-IL-4Ra-Stat6 axis (78). Lysozyme-processed and non-processed *Ruminococcus gnavus* (a lysozyme-sensitive bacterium) induce distinct immune responses in mice, indicating that lysozyme regulates the pro-inflammatory or anti-inflammatory effects of lysozyme-sensitive bacteria (78).

**Figure 2:**
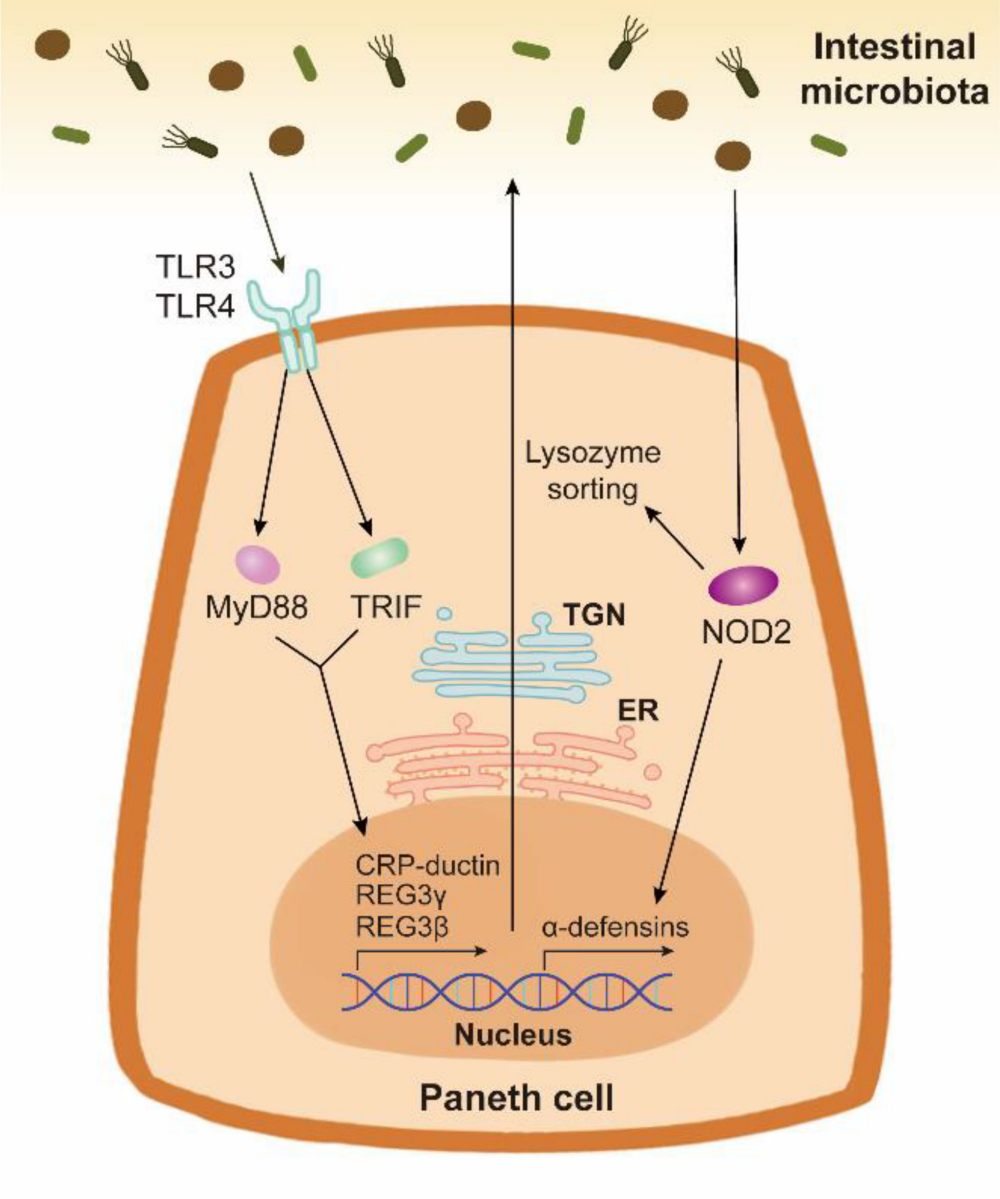
The AMP expression and secretion stimulated by the intestinal bacteria in Paneth cells. Paneth cells can sense the intestinal bacteria through pattern recognition receptors TLR and NOD2. TLR/MyD88 signaling and TLR/TRIF signaling activate the expression and release of AMPs such as CRP-ductin, REG3γ, and REG3β. Bacterium-stimulated NOD2 activation in Paneth cells contributes to the expression of α-defensins. The synthesis and packing of AMPs are respectively mediated by ER and TGN in Paneth cells. NOD2 also promotes lysozyme sorting, thus facilitating the release of lysozyme.

PCs have been confirmed to sense intestinal bacteria directly *via* cell-autonomous MyD88-dependent toll-like receptor (TLR) activation to induce the expression of AMPs (**Figure 2**) (79). Toll-IL-1 receptor domain-containing adaptor molecule 1 (TICAM1, also known as TRIF), another downstream protein of TLR, also increases AMP expression under homeostatic condition (80). These findings indicate that the TLR/MyD88 and TLR/TRIF signalings in PCs may explain why specific pathogen-free mice possess more PCs than germ-free mice (81). Nucleotide binding oligomerization domain containing 2 (NOD2), another pattern recognition receptor, also promotes AMP expression (**Figure 2**). In Caco-2 cells and the ileum of Crohn’s disease patients, NOD2 disruption decreases the gene expression of α-defensin, but not that of lysozyme (82, 83). However, the secretion of lysozyme is affected by NOD2. Lysozyme sorting is a lysozyme-specific process facilitating its secretion (84). After sensing intestinal bacteria, NOD2 promotes lysozyme sorting by recruiting LRRK2, RAB2A, and RIP2 (84, 85). HD5 overexpressing in NOD2-knockout mice can effectively enhance the bactericidal activity and reduce the susceptibility to *Helicobacter hepaticus* infection (86).

PC degranulation is a key event mediating AMP secretion, which can be induced by cholinergic agonists, bacteria, bacterial cell surface antigens, and cytokines such as IL-17, IL-22 and interferon (IFN)-γ (64, 66, 87, 88). Notably, in the intestinal organoids, PC degranulation can be induced by apical exposure (*via* microinjection) of lipopolysaccharide (LPS) or *Salmonella typhimurium*, but not by LPS in culture media (87). PC degranulation is usually accompanied by the increased cytosolic Ca2^+^ level (66, 89). After degranulation, PCs rapidly replenish their granules in response to subsequent stimulation (87).

## ISC niche support

As mentioned above, PCs are derived from ISCs through multiple regulation. Actually, PCs also serve as a supporter in ISC niche. Using individual leucine rich repeat containing G protein-coupled receptor 5 (Lgr5) stem cells, Sato et al. (34) established the small intestinal organoids containing PCs but not mesenchymal niche. The close physiological relationship between PCs and ISCs is demonstrated by co-culturing PCs and Lgr5 stem cells. The existence of PCs significantly enhances the formation efficiency of organoids (90). *In vivo* study has indicated that PC loss leads to a decrease in the expression of ISC marker olfactomedin 4 (*Olfm4*) (29). The fact that colonic organoid establishment requires the supplementation of Wnt3a into medium further confirms ISC-supporting role of PCs (91). cKit, as a tyrosine kinase receptor, labels PCs rather than ISCs in the small intestine (92). A subset of colonic goblet cells marked by cKit are adjacent to Lgr5 stem cells. Blocking Notch signaling induces the increase in cKit^+^ cell number in colon of mice, which is consistent with the above-mentioned observation of PCs in ileum (92). The co-culture of cKit^+^ cells and colonic Lgr5 stem cells promotes organoid formation *ex vivo*, suggesting a similar ISC-supporting role of cKit^+^ cells in colon (92). Stem cell factor (SCF), a ligand of cKit, is highly expressed in the small intestinal crypts under DSS treatment, and after the activation of SCF/cKit signaling in PCs, the co-culture of PCs and ISCs further enhances ISC functions (93). Numerous studies have demonstrated that IL-22 also improves ISC functions in both mice and organoids (94). IL-22Ra1 signaling in PCs is required for organoid growth under IL-22 treatment, suggesting that the beneficial effect of IL-22 on ISCs depends on PCs (33). In contrast, a recent study of the optimized human intestinal organoids has shown that IL-22 inhibits the growth of human intestinal organoids and boosts PC number and functions (95), which indicates that IL-22 signaling should be cautiously controlled. These findings imply that PCs facilitate the function of ISCs under both homeostasis and injury conditions.

**Figure 3:**
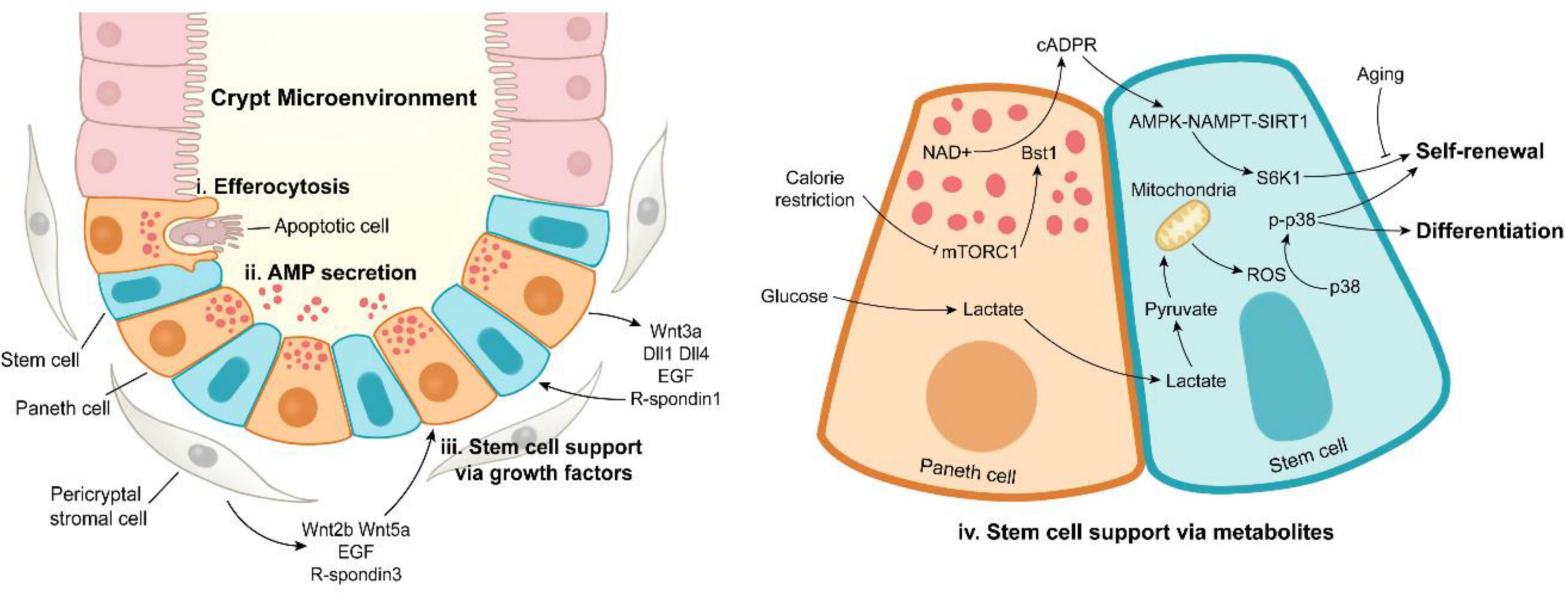
Paneth cell functions under intestinal homeostasis. Paneth cells maintain the stabilization of crypt microenvironment *via* efferocytosis of apoptotic cells (i), antimicrobial peptide secretion (ii), stem cell support by releasing growth factors (iii) or metabolites (iv). In addition to Paneth cells, pericryptal stromal cells are another important source of growth factors.

The ISC niche is comprised of epithelial niche and mesenchymal niche (2). Gene expression profiling reveals that epithelial niche characterized by PCs provides Wnt3, Dll4, epidermal growth factor (EGF), TGF-α, and R-spondin1 for ISC niche (90). Notably, the ISC-supporting role of PCs might be different in murine and human since the first emergence of PCs in murine is at 7-10 day after birth and that in human is before birth. The formation of intestinal organoids at the absence of mesenchymal niche highlights the importance of epithelial niche. Pericryptal stromal cells are major sources of Wnt2b, Wnt5a, EGF, and R-spondin3 in mesenchymal niche, and enteric glia cells secrete proEGF (EGF precursor form) to promote the intestinal regeneration (**Figure 3**) (96–100).

ISC markers *Lgr5* and *Ascl2* are target genes of Wnt signaling (101, 102). The transfer of Wnt signaling needs direct contact between PCs and ISCs through the membrane receptors composed of frizzled protein and low-density lipoprotein receptor-related protein 5/6 (LRP5/6) (103). The aged PCs exhibit the weakened ISC-supporting role due to the deficient Wnt3 supply (104) and the excessive release of Notum (105), an extracellular Wnt inhibitor. Although both PCs and mesenchymal components provide Wnt ligands, the two sources of Wnt signalings are redundant for ISC functions (106). Wnt3 deficiency has no effect on ISC maintenance in mice, but Wnt3 is required for organoid formation, implying a compensatory role of mesenchymal niche for ISC functions (106). In addition, the disruption of Wnt ligands secreted by PCs and myofibroblasts had no influence on ISC functions, suggesting the complexity of the ISC regulation (107). As an enhancer of Wnt signaling, R-spondin directly acts on Lgr5 to boost Wnt signaling activity, and it is necessary for Wnt ligand-mediated ISC maintenance (108). R-spondin3 has a more powerful effect on Wnt activation and organoid growth than R-spondin1 (98).

In addition to Wnt signaling, Notch signaling also plays a positive role in ISC niche. Notch inactivation leads to a reduction in Lgr5 stem cell number and *Olfm4* expression level (a Notch-regulated gene, another ISC marker) (42, 46). Notch ligands are transferred only to adjacent cells *via* cell-cell contact, and the transfer condition of Notch ligands is more strict than that of Wnt ligands (2). The expression of *Dll4* is confirmed in PCs, whereas that of *Dll1* is still under controversy. Mice with lacZ inserted at the *Dll1* locus exhibit β-galactosidase expression in the small intestinal lysozyme positive cells (45). By contrast, Dll1/lysozyme double fluorescent staining reveals that there is no Dll1 expression in lysozyme positive cells (109). In addition, high-level radical fringe proteins in PCs can increase Notch ligands on PC surface, thus elevating the number of adjacent Lgr5 stem cells (110). These findings further confirm that PCs promote ISC maintenance *via* Notch signaling. Notably, after PC ablation, enteroendocrine and tuft cells emerge at crypt bottoms, thus promoting ISC stabilization by providing Notch ligands, suggesting the flexibility of intestinal epithelium for ISC maintenance (111).

EGF signaling is responsible for regulating cell proliferation rather than ISC maintenance through EGF receptor (*EGFR*) highly expressed in ISCs (112). In ISC niche, EGF is secreted by PCs, enteric glia cells, and pericryptal stromal cells (*via* extracellular vesicles) (90, 96, 100). *EGFR* deletion leads to cell-cycle exit by disturbing MAPK/ERK signaling in organoids (113). Apart from MAPK/ERK signaling, PI3K/AKT signaling (a downstream signaling of EGFR) is also down-regulated after EGFR inhibition (113), but its role in ISC support requires further investigation. However, EGF signaling must be controlled discreetly, since ablation of leucine rich repeats and immunoglobulin like domains 1 (*Lrig1*), an ISC marker and negative regulator of EGFR, induces ISC expansion or even duodenal adenomas and superficially invasive carcinomas in mice (112, 114).

The ISC-supporting role of PCs is also achieved by metabolic regulation (**Figure 3**). Calorie restriction inhibits the activity of mammalian target of rapamycin complex 1 (mTORC1) to induce the expression of bone stromal antigen 1 (Bst1) converting NAD^+^ into cyclic ADP ribose (cADPR) in PCs (115). Once released, PC-derived cADPR paracrinically acts on ISCs and dramatically enhances their functions (115). A subsequent study has reported that calorie restriction-induced cADPR in PCs activates AMPK-Nampt-Sirt1 axis in ISCs to deacetylate S6K1, thus enhancing phosphorylation of S6K1 by mTORC1, eventually boosting ISC functions (116). Surprisingly, aging eliminates the calorie restriction-induced expansion of ISCs, which is associated with decreased number of PCs (117). In PCs, glycolysis provides lactate for ISCs, and the latter converts lactate into pyruvate, thus triggering mitochondrial oxidative phosphorylation (OXPHOS). OXPHOS-derived reactive oxygen species (ROS) facilitates p38 phosphorylation, thereby enhancing ISC function and differentiation (118). These studies emphasize the metabolic support effect of PCs on ISCs, and the metabolic interaction between PCs and ISCs may be an interesting topic to be further explored.

## Crypt microenvironment stabilization

It is generally accepted that the intestinal crypt is a sterile microenvironment. The intestinal epithelium is covered with a chemical layer mainly consisting of mucus (from goblet cells) and AMPs (primarily from PCs) (1). Although mucus is penetrable to bacteria, mucus in combination with AMPs maintains the sterility of intestinal crypts (119), suggesting the importance of PCs for crypt microenvironment stabilization. PC disruption potentially results in bacterial colonization in crypts or even bacterial penetration into mucosa in the intestine. Gene ablation such as X-linked inhibitor of apoptosis protein (*XIAP*), autophagy-related gene 7 (*ATG7*), and *caspase-8* leads to the loss and abnormality of PCs as well as subsequent bacterial colonization in crypts (39, 120–123). Under the condition of unimpaired intestinal epithelium, PC disruption exacerbates the bacterial translocation to mesenteric lymph nodes (MLN) and other sites such spleen and liver (79, 124). These findings suggest that crypts are in a contaminated state under PC disruption.

The bacterial colonization in crypts may trigger ISC apoptosis. Chemotherapy such as 5-fluorouracil and doxorubicin (Dox) treatments can induce intestinal bacterial translocation and the apoptosis of PCs and ISCs in mice (125). Antimicrobial administration rescues Dox-induced ISC loss (126), implying that sterile crypts protect ISCs from excessive apoptosis. Based on these findings, it could be speculated that PC disruption-mediated bacterial colonization in crypts might bring about ISC apoptosis and loss. This speculation is confirmed by one report that *ATG7* knockout causes PC dysfunction and bacterial colonization in crypts, thus potentially inducing ISC apoptosis in mice, while *ATG7* knockout has no effect on organoid growth *ex vivo* (127). Antimicrobial treatment prevents ISC loss in *ATG7* knockout mice (127), further demonstrating the indispensable role of PCs in crypt microenvironment stabilization.

The intestinal epithelium possesses high turnover rate with a large number of apoptotic cells rapidly supplemented to sustain optimal barrier and absorptive functions (1). CD95, a member of TNF receptor family, is expressed on the basolateral surface of intestinal epithelial cells (IECs), and it can trigger cell apoptosis in the intestine (128). PCs secrete CD95 ligand to drive apoptosis of IECs, suggesting the potential role of PCs in regulating epithelial integrity (129). The apoptotic cells at villus tip are shed into the intestinal lumen (130), and other apoptotic cells on the intestinal epithelium are engulfed by efficient phagocytes (macrophages and dendritic cells) and inefficient phagocytes (IECs) (131, 132). The accumulation of excessive apoptotic cells engenders a pro-inflammatory microenvironment (133, 134). Recently, an unanticipated phagocytic role of PCs has been reported (**Figure 3**) (21). PCs mediate the uptake of neighboring apoptotic cells *in vivo* and *ex vivo*, and PC deletion leads to apoptotic cell increase and efferocytosis disappearance in crypts under homeostasis and irradiation conditions (21), suggesting a novel apoptotic cell phagocytic function by PCs, which needs to be further explored.

## End of PC life: Death and dedifferentiation

**Figure 4:**
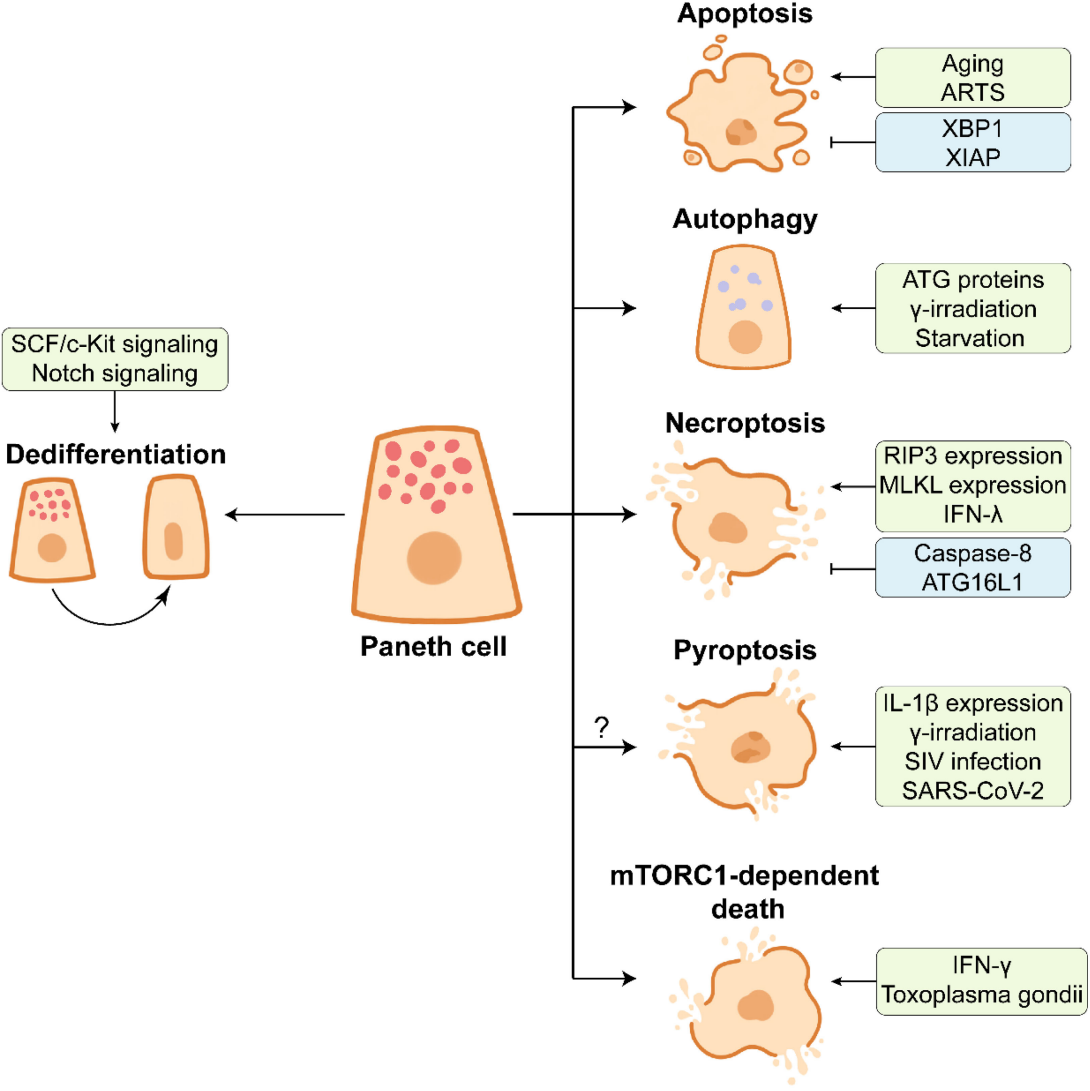
The end of Paneth cell life. Paneth cells dedifferentiate into stem-like cells by activating SCF/c-Kit signaling or Notch signaling in response to the intestinal injury, which facilitates intestinal proliferation and repair. Aging or ARTS-expressing Paneth cells undergo apoptosis under the intestinal homeostasis. XBP1 and XIAP have opposite effects on apoptosis of Paneth cells. Paneth cells can remove the damaged organelles and proteins by autophagy, which contributes to the maintenance of cellular homeostasis. ATG proteins, γ-irradiation, and starvation are associated with the activation of autophagy in Paneth cells. The necroptosis of Paneth cells is frequently observed in the inflamed small intestine. The necroptosis of Paneth cells is promoted by highly expressed RIP3 and MLKL and pro-inflammatory cytokine IFN-λ, and it is inhibited by caspase-8 and ATG16L1. Multiple factors such as high IL-1β expression in Paneth cells, γ-irradiation, SIV infection, and SARS-CoV-2 infection may trigger the pyroptosis of Paneth cells. In Toxoplasma gondii infection, IFN-γ induces mTORC1-dependent death of Paneth cells.

PCs possess prolonged lifespan, relative to enterocytes, and they undergo spontaneous cell death in the small intestinal crypts (**Figure 4**) (135). After approximately one-month hard work, PCs end their lives *via* apoptosis which represents an immunologically silent form of programmed cell death (PCD), as demonstrated by the abundant expression of pro-apoptotic protein ARTS (apoptosis-related protein in the TGF-β signaling pathway) in PCs (136). *ARTS* deficiency prevents apoptosis of PCs and increases the number of PCs (136). In addition, PC apoptosis can be accelerated in some cases. Gene ablation of *XIAP* and *XBP1* (X-box-binding protein 1) and ischemia-reperfusion cause apoptotic PC loss (39, 124, 137). PC disruption in DTX-treated PC-DTR mice leads to robust apoptosis of PCs (24). However, dithizone selectively induces the degeneration to destroy PCs without triggering inflammation (22).

Considering that PCs possess a prolonged lifespan in the intestine, autophagy is essential for the removal of the broken organelles and redundant proteins from PCs (9). γ-irradiation and starvation treatments significantly activate autophagy of PCs and up-regulate the expression of LC3 II (138–140). ATG family proteins play an important role in PC autophagy. The abnormality of ATG family proteins such as autophagy related 16 like 1 (ATG16L1) and ATG7 leads to the defects in AMPs expression in PCs from mice and Crohn’s disease patients (31, 141, 142).

Necroptosis is a pro-inflammatory type of PCD, accompanied by the release of immuno-stimulating DAMPs (damage-associated molecular patterns) such as mtDNA and ATP (143). In addition to apoptosis, necroptosis frequently occurs in PCs under inflammatory conditions. PCs from human and mouse exhibit an abundance of receptor-interacting protein 3 (RIP3), a key regulator of necroptosis, which renders PCs susceptible to necroptosis (120). Crohn’s disease and mouse intestinal inflammation are closely associated with strong necroptosis of PCs (120, 144), and PC necroptosis is further associated with gene ablation and cytokines. *Caspase-8* deletion leads to necroptotic PC loss (120). *ATG16L1* deficiency-induced disruption of mitochondrial homeostasis triggers PC necroptosis in response to TNF-α challenge (145). In addition, pro-inflammatory cytokine IFN-λ induces PC necroptosis *via* caspase-8/mixed lineage kinase domain-like (MLKL) signaling (146). The elevated level of IFN-γ induced by Toxoplasma gondii infection results in PC death which is mTORC1-dependent and different from canonical PCD (147). Necroptotic PC loss can be rescued by *RIP3* knockout or Necrostatin-1 (an inhibitor of necroptosis) administration (39, 148). Whether PC necroptosis is associated with the intestinal inflammation still remains to be further investigated.

Pyroptosis, another form of PCD, may also occur during the end of PC life. Pyroptosis is mediated by caspase-1 protease promoting IL-1β maturation (143). The expression of IL-1β is much higher in PCs than in lamina propria under intestinal homeostasis (149). The activation of caspase-1 and IL-1β as well as the release of IL-1β are observed in PCs at day 7 post γ-irradiation, suggesting the potential pyroptosis in PCs (138). A large amount of IL-1β is produced by PCs before the type i IFN response, thus impairing epithelial barrier in early simian immunodeficiency virus (SIV) infection (149). It should be noted that the expression of NLRP3, a key component of NLRP3 inflammasome facilitating pyroptosis, is increased in PCs from SARS-CoV-2-infected rhesus macaques (150). More research needs to be conducted so as to understand the importance of PC pyroptosis in the intestine.

As mentioned above, PCs are differentiated from ISCs through a series of lineage commitments, and PCs can dedifferentiate into multipotent stem cells in some injury cases (**Figure 4**). In the DSS-induced intestinal inflammation, the differentiated and post-mitotic PCs dedifferentiate into stem-like cells, thus promoting tissue regenerative response by activating the SCF/cKit signaling and its downstream PI3K/Akt and Wnt signalings (93). The functions of Notch signaling in PC maintenance has been discussed above, and another function of Notch signaling in PCs is to facilitate PC dedifferentiation. Notch signaling activation drives Defa4-labelled PCs to dedifferentiate into ISCs, which benefits the intestinal regeneration in Dox-induced injury model (151). Irradiation activates Notch signaling to endow PCs with stem cell features (152). Surprisingly, the intact intestinal organoids are established based on the isolated PCs with stem cell features, implying the plasticity of dedifferentiated PCs (152). These findings suggest that the end of PC life is a complicated process which is affected by many factors.

## Conclusion

With the increasing attention to the multiple functions of PCs, a brief summary of materials and methods used for PC study is badly needed. PCs are devoted to maintaining the intestinal homeostasis during their entire lives. PC, a long-lived intestinal epithelial cell, is differentiated from adjacent Lgr5 positive ISCs, and it migrates downward to the bottom of crypts after maturation. The presence of abundant granules in the cytoplasm is the most striking feature of PCs, which is used to identify the presence of PCs in the intestine. Although it has been 150 years (1872–2022) since the first identification of PCs, the multiple functions of PCs in the intestine are not completely understood. In recent years, the novel functions of PCs have been continuously reported. In the intestine, PCs endow the intestinal crypts with sterile environment by secreting AMPs, and they support ISC functions by providing growth factors and metabolites. Apoptotic cells in crypts can be promptly engulfed by PCs, thus preventing inflammation. At the end of life, PCs undergo canonical PCD and heroic dedifferentiation. Considering that PCs are located at the crypt bottom above the lamina propria, it is worth exploring the influence of pro-inflammatory PC death on the intestinal inflammation in future work.

## Author contributions

CC (conceptualization: lead; writing - original draft: lead; visualization: supporting; writing - review & editing: supporting). FW (visualization: lead). YZ (writing - original draft: supporting). HW (writing - review & editing: supporting). JP (funding acquisition: lead; writing - original draft: supporting; writing - review & editing: lead). All authors contributed to the article and approved the submitted version.
